# The Computational Fluid Dynamics Analyses on Hemodynamic Characteristics in Stenosed Arterial Models

**DOI:** 10.1155/2018/4312415

**Published:** 2018-03-14

**Authors:** Yue Zhou, Chunhian Lee, Jingying Wang

**Affiliations:** ^1^School of Aeronautic Science and Engineering, Beihang University, Beijing 100191, China; ^2^School of Energy and Power Engineering, Shandong University, Jinan 250061, China

## Abstract

Arterial stenosis plays an important role in the progressions of thrombosis and stroke. In the present study, a standard axisymmetric tube model of the stenotic artery is introduced and the degree of stenosis *η* is evaluated by the area ratio of the blockage to the normal vessel. A normal case (*η* = 0) and four stenotic cases of *η* = 0.25, 0.5, 0.625, and 0.75 with a constant Reynolds number of 300 are simulated by computational fluid dynamics (CFD), respectively, with the Newtonian and Carreau models for comparison. Results show that for both models, the poststenotic separation vortex length increases exponentially with the growth of stenosis degree. However, the vortex length of the Carreau model is shorter than that of the Newtonian model. The artery narrowing accelerates blood flow, which causes high blood pressure and wall shear stress (WSS). The pressure drop of the *η* = 0.75 case is nearly 8 times that of the normal value, while the WSS peak at the stenosis region of *η* = 0.75 case even reaches up to 15 times that of the normal value. The present conclusions are of generality and contribute to the understanding of the dynamic mechanisms of artery stenosis diseases.

## 1. Introduction

At present, arterial stenosis has been clinically regarded as playing a key role in some cardiovascular or cerebrovascular diseases, such as atherosclerotic plaque, thrombosis, and stroke [1, 2]. As the artery narrows, blood flow patterns and the vessel wall shear stress (WSS) will change greatly, which contributes to both thrombus formation and plaque cap rupture. However, it is difficult to directly measure hemodynamic factors of the diseased artery *in vivo*. CFD has become a very useful technique for predicting detailed information on human blood flows including flow patterns, pressures, and WSS [3].

Steinman et al. [4] simulated blood flows in both concentrically and eccentrically stenosed carotid bifurcation models to study geometrical effects on flow patterns. They proposed that the stenosed carotid bifurcation geometry may provide more specific indicators for vulnerable plaques. Long et al. [5] calculated blood flows in both axisymmetrical and asymmetrical stenosis models with three different area reductions and found that in axisymmetrical models, the poststenotic blood flow is more sensitive to variations of the stenosis degree than in asymmetrical cases. Khader et al. [6] used CFD to study the blood flow in a simple vessel model of 66% eccentric stenosis generated from Doppler scan and demonstrated that blood flows changed dramatically with the increase in the severity of stenosis at throat region, which can cause a growth in velocity and WSS. Sui et al. [7] employed CFD to investigate the WSS, velocity, and pressure distributions in areas near carotid artery plaques and analyzed the relative change of hemodynamic parameters with different stenosis degrees.

Although many arterial stenosis cases have been investigated by the CFD technique, there remain several problems. On one hand, in some previous simulation works, the blood was treated to be Newtonian, while the real blood is non-Newtonian with rheological effects [8, 9]. On the other hand, most stenosis models used in prior CFD studies were always patient specific [10, 11], which could illustrate some unique flow characteristics in diseased arteries, but the conclusions may be lacking in theoretical generalization. Therefore, the present study introduces a standard axisymmetrical cylindrical tube model as the stenosed artery and defines the corresponding degree of stenosis. Then, the blood flows through arterial models of different stenosis degrees are solved by CFD, respectively, with the Newtonian and a non-Newtonian constitutive model (the Carreau model [12]) for comparison. Finally, the hemodynamic characteristics of flow patterns, pressure drops, and WSS distributions are investigated quantitatively.

## 2. Stenosed Artery Model

**Figure 1:**
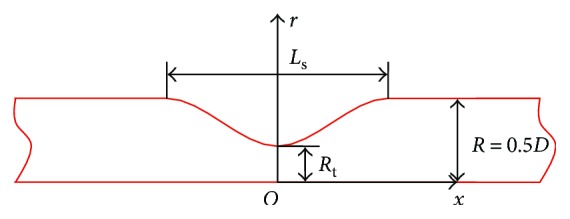
Axisymmetrical tube model of the stenosis artery.

The stenosed artery is simplified into a parameterized cylindrical tube model and currently only the axisymmetric stenosis case is investigated for preliminary study. As shown in Figure 1, *x* and *r* represent axial and radial coordinates, respectively; *R* and *D* are the tube radius and diameter, respectively; *R*_t_ is the radius of the stenosis throat; and *L*_s_ is the length of the stenosis region. A cosine curve is selected to describe the stenosis shape. The degree of stenosis *η* is defined as the ratio of the blockage area to the normal vessel cross-sectional area and is as follows:

(1) $$\begin{array}{c} \eta =\frac{\pi R^{2}-\pi R_{t}^{2}}{\pi R^{2}}=1-{\left(\frac{R_{t}}{R}\right)}^{2}. \end{array}$$

## 3. Numerical Schemes and Validation

Assuming laminar flow and neglecting the influence of body force, the arterial blood flow can be described by the incompressible viscous Navier-Stokes (N-S) equations as follows:

(2) $$\begin{array}{c} \nabla \cdot U=0,\\ \frac{\partial \rho U}{\partial t}+\nabla \cdot \left(\rho UU\right)=-\nabla p+\nabla \cdot \tau , \end{array}$$

where **U** is the flow velocity vector; *ρ* is the blood density, with a value of 1050 kg/m^3^; and *p* is the pressure. **τ** is the viscous stress tensor and is expressed as follows:

(3) $$\begin{array}{c} \tau =\mu \left[\nabla U+{\left(\nabla U\right)}^{T}\right], \end{array}$$

where *μ* is the dynamic viscosity. If the blood is treated as a Newtonian fluid, the viscosity *μ* will be a constant. However, the real blood is a multicomponent mixture consisting of blood cells and plasma, which behaves rheologically [13]. In this paper, the non-Newtonian effects of blood are calculated by the Carreau model as follows:

(4) $$\begin{array}{c} \mu =\mu_{\infty }+\left(\mu_{0}-\mu_{\infty }\right){\left[1+{\left(\lambda \gamma \right)}^{2}\right]}^{\left(n-1\right)/2}. \end{array}$$

**Figure 2:**
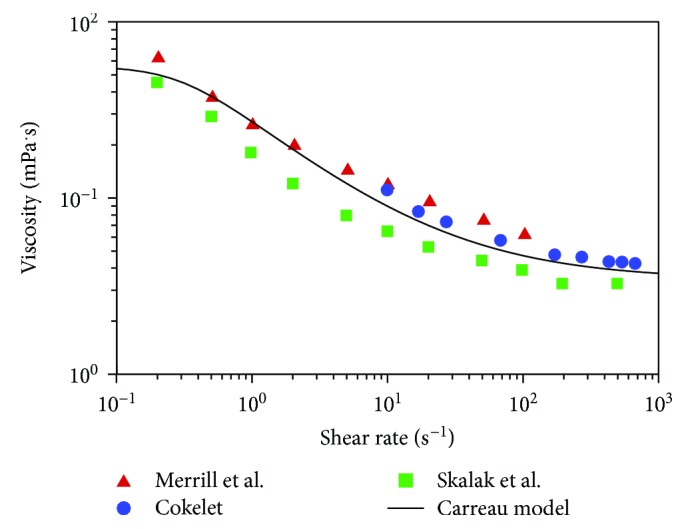
Comparison of blood viscosity given by the Carreau model with experimental data.

Equation (4) is an empirical expression, and *γ* is the local shear rate. There are *μ*_∞_ = 0.00345 Pa · s (the plasma viscosity), *μ*_0_ = 0.056 Pa · s, *λ* = 3.313 s, and *n* = 0.3568.  Figure 2 compares the blood viscosity calculated by the Carreau model with experimental data from various sources (Merrill et al. [14]; Cokelet [15]; Skalak et al. [16]), which demonstrate that the Carreau model used in this work has a reasonable accuracy.

**Figure 3:**
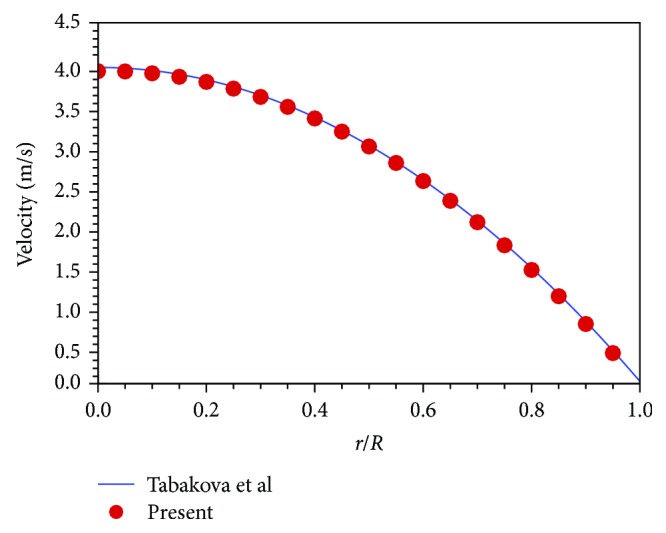
Validation of the present numerical scheme.

When implementing CFD simulations, (2), (3), and (4) are solved by the semi-implicit method for pressure-linked equations (SIMPLE) [17], which has been widely used to compute incompressible viscous flow problems. In the SIMPLE algorithm, the pressure and momentum equations are both discretized in second-order schemes to give accurate viscous blood flow results. A case of non-Newtonian tube blood flow calculated by Tabakova et al. [18] is employed to validate the present numerical scheme with the Carreau model. In this case, the tube radius is *R* = 0.0031 m, and the flow rate is *Q* = 5.98 × 10^−5^ m^3^/s. The blood density is set to be *ρ* = 1000 kg/m^3^.  Figure 3 shows that the present results are very consistent with the data of Tabakova et al., which validate that the present numerical scheme can solve the tube blood flow accurately by the Carreau model.

## 4. Grids and Boundary Conditions

**Figure 4:**
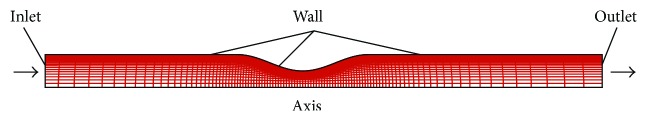
Computational grids and boundary conditions of the *η* = 0.75 case.

Five cases with different stenosis degrees, *η* = 0, 0.25, 0.5, 0.625, and 0.75, are simulated, respectively, by using the Newtonian and Carreau models for comparison in this paper, where *η* = 0 represents the normal vessel without stenosis as the base case. For all cases, the radius of the arterial vessel is fixed at *R* = 5 mm (*D* = 10 mm) and *L*_*s*_ = 4*R*. Computational grids of all cases are structural quadrangle elements and distributed nonuniformly. Close to the vessel wall and the stenosis, grid nodes are denser than other regions, so that the simulation can capture and recognize the flow patterns and hemodynamic characteristics clearly.  Figure 4 shows the computational grids of the *η* = 0.75 case.

For all cases, boundary condition settings are the same. As illustrated in Figure 4, the left boundary is set to be the blood flow inlet, the top is the wall, the bottom is the axis, and the right is outlet. The blood flow rate of the inlet is constant with a value of *Q*_b_ = 0.465 L/min (the mean velocity *U*_m_ = 0.0986 m/s). Under this flow rate, the Reynolds number is 300, which is calculated by using the vessel diameter and plasma viscosity.

## 5. Results and Discussions

This work focuses on analyzing the CFD results of flow patterns, pressure drops, and WSS distributions, which have the profound influence on the progression and diagnosis of artery stenosis in clinical settings.

### 5.1. Flow Patterns

**Figure 5:**
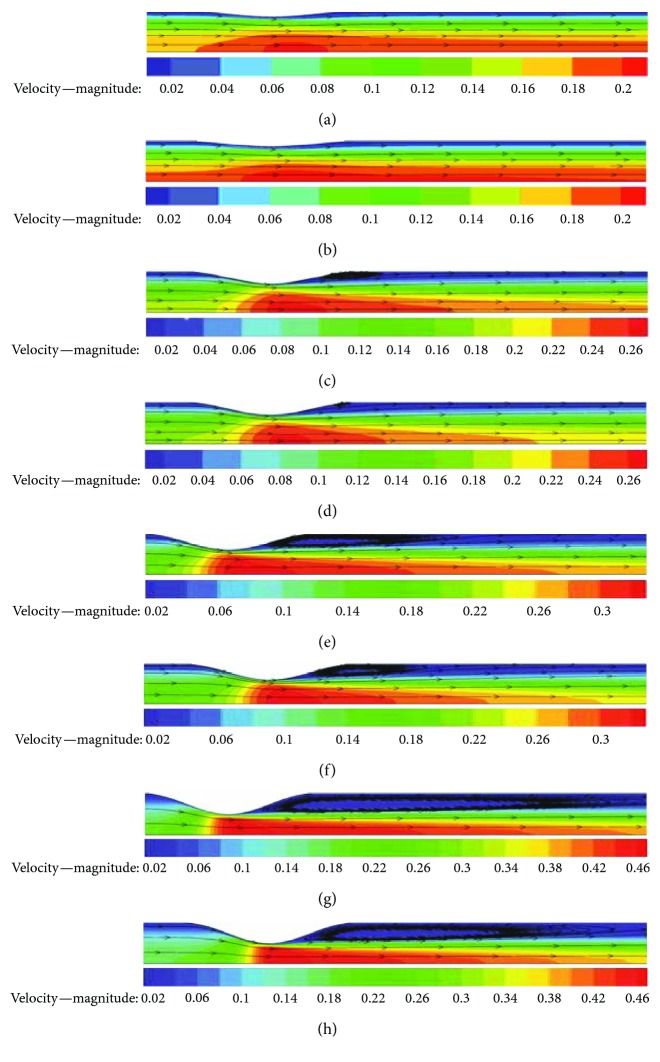
Flow patterns: (a) Newtonian, *η* = 0.25; (b) Carreau, *η* = 0.25; (c) Newtonian, *η* = 0.5; (d) Carreau, *η* = 0.5; (e) Newtonian, *η* = 0.625; (f) Carreau, *η* = 0.625; (g) Newtonian, *η* = 0.75; (h) Carreau, *η* = 0.75.

Blood flow patterns have been considered to be specific indicators of vulnerable plaques [4]. Once stenosis happens in the artery, flow patterns can change greatly. A very important phenomenon is that there will be flow separation vortexes in the poststenotic regions of severe stenosis cases, which is validated by the present CFD results.  Figure 5 demonstrates that for both the Newtonian and Carreau models, the more severe the stenosis is, the bigger the separation vortex becomes. When the stenosis degree relieves to 0.25, the vortex disappears completely. Another notable phenomenon in Figure 5 is that for all cases, there is a poststenotic slow velocity region (blue-colored region) and this region enlarges as the stenosis becomes severe. In this region, the mass transportation is weak and the WSS is also small, where thrombosis is most likely to happen in clinical settings.

**Figure 6:**
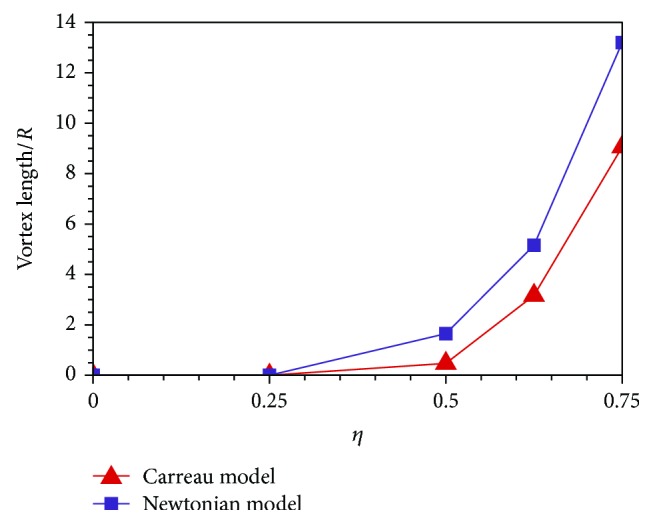
Separation vortex length.

Figure 6 plots the vortex lengths nondimensionalized by the vessel radius *R* for cases of different stenosis degrees. As the vessel narrows gradually, the separation vortex lengths of the Newtonian and Carreau models both increase exponentially. When *η* = 0.75, the non-Newtonian vortex length can reach up to nine times that of the vessel radius. Figure 6 also shows that the vortex length of the Newtonian model is longer than that of the Carreau model, which substantiates that the Carreau model predict higher viscous dissipation than the Newtonian model. Therefore, the use of the Newtonian model for blood flow simulation should be cautious.

### 5.2. Pressure

**Figure 7:**
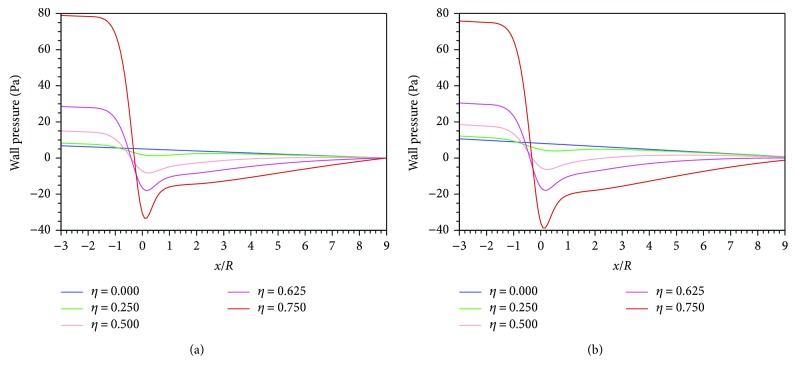
Axial wall pressure distributions of all stenosis cases: (a) Newtonian; (b) Carreau.

**Figure 8:**
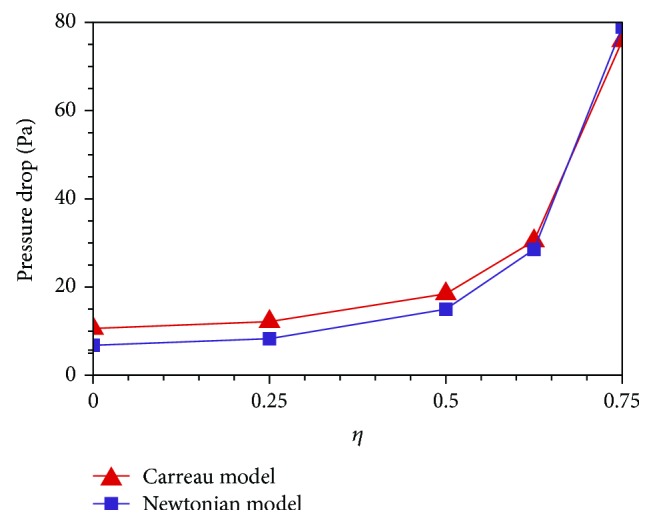
Pressure drops from *x* = −3*R* to *x* = 9*R* of all stenosis cases.

Figure 7 shows the axial wall pressure (WP) distributions of all cases from *x* = −3*R* to *x* = 9*R* predicted, respectively, by the Newtonian and Carreau models. The zero pressure point is set at *x* = 9*R*. For both models, the axial WP reduces linearly without stenosis, while it varies tortuously around the stenosis. The trend can be elucidated with the variation of the blood flow velocity. For the normal case (*η* = 0), the blood flow is fully developed with the unchanged velocity profile and the pressure gradient only needs to balance with the constant WSS along the axis. Therefore, the axial pressure reduces linearly. Once the stenosis happens, the velocity increases and the pressure decreases with the stenosis severity by the Bernoulli's theorem.  Figure 8 illustrates that for both models, the pressure drop of *η* = 0.75 case is nearly 8 times that of the normal case. Therefore, artery stenosis can cause high blood pressure, which has been explained in [19]. For the Newtonian flow, if the flow rate *Q* is constant, there is

(5) $$\begin{array}{c} \frac{\delta \left(\Delta p\right)}{\Delta p}=-4\frac{\delta D}{D}, \end{array}$$

where δ is the differential operator, Δ*p* is the pressure drop, and *D* is the tube radius. Equation (5) means that the variations of pressure drop and radius have opposite trends. According to (5), when the radius decreases, the pressure drop necessarily rises. The present CFD results validate the same for the non-Newtonian blood flow.

### 5.3. WSS

**Figure 9:**
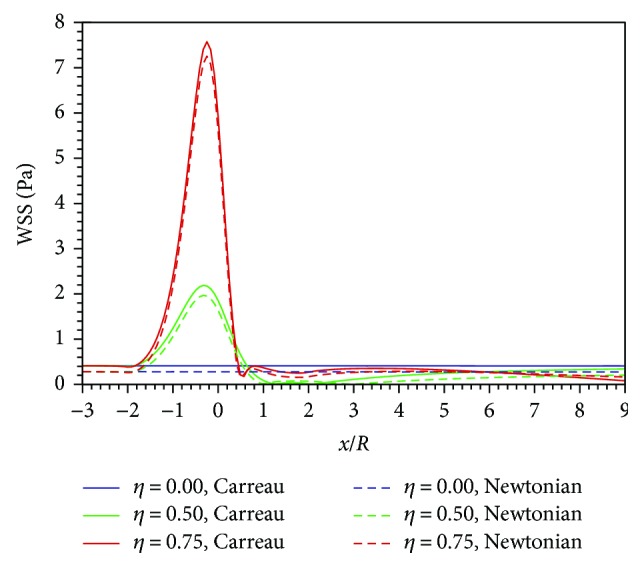
WSS distributions along the axis of all cases.

Existing studies always focus on analyzing the WSS characteristics of stenosis diseases. High WSS in the partially occluded vessel has been known to activate platelet-platelet binding events that play a very important role in thrombosis [4]. This work also predicts the WSS distributions of all stenosis and normal cases, respectively, by the Newtonian and Carreau models.  Figure 9 displays the WSS distributions from *x* = −3*R* to *x* = 9*R* along the axis of the normal (*η* = 0), *η* = 0.5, and *η* = 0.75 cases for both models. Under each stenosis degree, the WSS of the Carreau model is a little greater than that of the Newtonian model but both have imilar trends. For two models, at the stenosis region (*x* = −2*R* to *x* = 2*R*), the stenosis WSS is remarkably higher than that of the normal case and WSS peaks both appear around the stenosed centre (*x* = 0). The WSS peak increases dramatically with the growth of the stenosis degree. When the stenosis degree rises to *η* = 0.75, the WSS peaks of both models reach up to about 7.5 Pa, almost 15 times that of the normal value (about 0.5 Pa). Therefore, artery stenosis indeed causes a remarkable WSS increase. In fact, according to Figure 5 and (3), the high-speed flow at the stenosis region results in a high WSS, and clinically, this region may be the fibrous cap in the vessel. The corresponding mechanical stress can contribute to plaque rupture and dislodged material [20].

## 6. Conclusions

Although the CFD technique has been widely used to investigate arterial stenosis, many previous studies solved the blood flows through unique patient-specific artery models, the conclusions of which may be the lack of generalization. In addition, some research employed only the Newtonian constitutive model for CFD simulation, which cannot reflect the rheological behaviour of blood. Therefore, the current study introduces a standard tube model of arterial stenosis and simulates tube blood flows, respectively, by the Newtonian and (non-Newtonian) Carreau models for comparison.

A normal (*η* = 0) and four diseased cases of different stenosis degrees, *η* = 0.25, 0.5, 0.625, and 0.75, are all computed with a constant Reynolds number, Re = 300. For the Newtonian and Carreau models, both results quantitatively show that the severe narrowing can cause the remarkable poststenotic flow separation and the length of separation vortex elongates exponentially as the tube contracts. However, the non-Newtonian vortex is shorter than the Newtonian vortex, because the viscous dissipation of the Carreau model is higher than that of the Newtonian model. The arterial stenosis accelerates the blood flow and causes the high blood pressure drop. For both models, the pressure drop of *η* = 0.75 case is nearly 8 times that of the normal value. The WSS of the Carreau model is a little greater than that of the Newtonian model. The artery narrowing leads to a high WSS increase at the stenosis region. For both models, the WSS peak of *η* = 0.75 case even reaches up to 15 times that of the normal.
